# Effectiveness of a new non-hydrogen peroxide bleaching agent after single use - a double-blind placebo-controlled short-term study

**DOI:** 10.1590/1678-7757-2016-0463

**Published:** 2017

**Authors:** Mozhgan Bizhang, Julia Domin, Gholamreza Danesh, Stefan Zimmer

**Affiliations:** 1 Universität Witten/Herdecke, Department of Operative and Preventive Dentistry, Witten, Germany; 2 Universität Witten/Herdecke, Department of Orthodontics, Witten, Germany

**Keywords:** Efficacy, Hydrogen peroxide, Double-blind study, Tooth bleaching, Side effects

## Abstract

Tooth whitening represents perhaps the most common aesthetic procedure in dentistry worldwide. The efficacy of bleaching depends on three aspects: bleaching agent, bleaching method, and tooth color.

**Objective:**

This *in vivo* study aimed to examine whitening effects on frontal teeth of the upper and lower jaws using an over-the-counter (OTC) non-hydrogen peroxide bleaching agent in comparison to a placebo after one single use.

**Material and methods:**

Forty subjects (25 female; 15 male) participated in this double-blind randomized placebo-controlled trial. The subjects were randomly allocated to two groups (n=20). The test group received the OTC product (iWhite Instant) and the placebo group received an identically composed product except for the active agents. Each subject was treated with a prefilled tray containing iWhite Instant or the placebo for 20 minutes. The tooth shade of the front teeth (upper and lower jaws) was assessed before (E_0), immediately after (E_1) and 24 h after treatment (E_2), using a shade guide (VITA classical). Statistical testing was accomplished using the Mann-Whitney U test (p<0.001). The dropout rate was 0%.

**Results:**

There were no significant differences at E_0 between placebo and test groups regarding the tooth color. Differences in tooth color changes immediately after (ΔE1_0) and 24 h after treatment (ΔE2_0) were calculated for both groups. The mean values (standard deviations) of tooth color changes for ΔE1_0 were 2.26 (0.92) in the test group and 0.01 (0.21) in the placebo group. The color changes for ΔE2_0 showed mean values of 2.15 (1.10) in the test group and 0.07 (0.35) in the placebo group. For ΔE1_0 and ΔE2_0 significant differences were found between the groups.

**Conclusion:**

In this short-term study, the results showed that a non-hydrogen peroxide bleaching agent has significant whitening effects immediately and 24 h after a single-use treatment.

## Introduction

According to representative data from the United Kingdom and from a study with Chinese urban population, the prevalence of tooth discoloration in 13-65 year-olds is approximately 50%^3^ ^,^ ^27^. Consequently, tooth whitening is perhaps the most frequently applied aesthetic procedure in dentistry.

The etiology of tooth discolorations is multi-causal and can result from individual behavior, diseases, injury and other exposures along with various physiological processes^1^. Professional cleaning of discolored teeth is a common procedure to remove the majority of extrinsic strains. Various bleaching techniques and products are used to remove intrinsic stains: In-office or power bleaching, home bleaching, and over-the-counter bleaching products.

Most bleaching products use hydrogen peroxide as active agent. However, bleaching treatments with peroxide may cause local adverse effects such as oral mucosa irritation, pulpal sensitivity, pulpitis or alteration of the enamel surface^14^. On the other hand, bleaching is a relatively safe procedure that predominantly causes severe adverse effects only at high hydrogen peroxide concentrations on hard tissue, soft tissue and restorative materials^15^. The European Scientific Committee on Consumer Products (SCCP)^4^ reported that the use of tooth whitening products containing >0.1 to 6.0% hydrogen peroxide or equivalent hydrogen peroxide-releasing substances is safe after consultation with a dentist.

On 31 October 2012, the EU Council Directive 2011/84/EU (amending EU Council Directive 76/768/EEC) specified that OTC tooth-whitening products and kits may only contain up to 0.1% hydrogen peroxide. However, this concentration is too low to have any noticeable effect on the color of teeth.

Legally, products that contain more than 0.1% hydrogen peroxide can only be sold to a dentist^24^. In January 2008, the SCCP recommended 6% hydrogen peroxide as a safe limit to use for home bleaching if supervised by a dentist^24^. Tooth bleaching based on non-peroxide systems are available as over-the-counter products in the form of gels, rinses, gums, dentifrices, whitening strips or paint-on films^10^ ^,^ ^15^ ^,^ ^24^. Auschill, et al.^5^ (2005) examined the efficacy and side effects of three different bleaching treatments: Whitestrips (OTC product, 5.3% hydrogen peroxide), Opalescence PF 10% (home bleaching, 10% carbamide peroxide) and Opalescence Xtra Boost (in-office bleaching, 38% hydrogen peroxide). The side effects were reversible and the test products had no harmful effect on the tooth surfaces. Additionally, the treatments were effective in removing intrinsic stains^4^, but given the actual legislation, such products are no longer allowed to be sold as OTC products without supervision of a dentist. Various studies investigated the effectiveness, safety and possible rebound effect of at-home and OTC agents. The most common side effects after bleaching were dental hypersensitivity and gingival irritation^18^. Moreover, the efficacy of products containing hydrogen peroxide is generally based on cumulative and repeated treatments; no instant or one-treatment efficacy has been reported. No clinical studies were found for non-peroxide bleaching products. Only one study examined a non-peroxide at-home bleaching product based on sodium chloride *in vitro* and observed deteriorating effects on dental enamel^20^.

A novel OTC bleaching agent based on nonperoxide gel was investigated in this study. It contains phthalimido peroxy caproic acid (PAP) and calcium lactate gluconate as active ingredients and has a high potential of oxidation with release of active oxygen. The gel should be placed on a ready-to-use tray. The calcium-lactate-gluconate is an implemented complex to remineralize and conserve dental hard tissue. A combination of bleaching and remineralization agents was efficient in reducing dental hypersensitivity^8^ ^,^ ^23^. Thus, the purpose of this randomized, double-blind, and placebo-controlled short-term study was to determine the efficacy and side effects of a novel OTC non-peroxide bleaching agent after a single-use application.

In this double-blind study, the primary goal was to test the hypothesis that there are significant differences in tooth color units (1-16 units) between a novel OTC non-peroxide bleaching and placebo immediately after a single-use application regarding the effectiveness of bleaching. The secondary goal was to determine the color change as well as the side effects between baseline and after 24 h plus between immediately and after 24 h bleaching.

## Material and Methods

### Ethical approval

Written informed consent was obtained from all participants, and all procedures of the study were clearly explained in advance. The study was conducted according to the Declaration of Helsinki principles for medical research involving human subjects. The study was approved by the ethical committee of Witten/Herdecke University, Germany (No. 26/2014), where the full trial protocol can be accessed. The study has the following German Clinical Trials Register number: DRKS00007636, date: 7 January 2015. The sample size was calculated using G*Power (version 3.0; http://www.psycho.uni-duesseldorf.de/abteilungen/aap/gpower3)^12^. The number of participants for the primary outcome was set at 20 *per* group to allow for dropout and to ensure an adequate power of 80% and an alpha error of 0.05 to detect a difference of one-shade guide units and 0.5 units of standard deviation between the two groups with an effect size of 1. Methods were applied in accordance with the approved guidelines.

### Study population

Forty volunteers (15 men and 25 women) with a mean age of 35.1±14.5 years participated in this study. The first 40 subjects from 50 volunteers from screening examination who were interested and agreed to participate and fulfilled the inclusion and exclusion criteria were asked to sign the consent form and enrolled in the study. The subjects received dental prophylaxis, then a single average shade score was calculated for each individual. This study was a double-blind randomized parallel study, and subjects were randomly assigned to one of the two groups, balancing for gender and baseline tooth color. Randomization was done with a sealed envelope system by a person who was not involved in the trial. The randomization was stratified according to four combinations of color shade and gender (female & average shade color darker than A3/female & average shade color brighter than A3/male & average shade color darker than A3/male & average shade color brighter than A3). The participants were given randomly generated treatment allocations within sealed opaque envelopes.

Test group (n = 20): iWhite Instant (Sylphar N.V., Deurle, Belgium) with bleaching agents;

Placebo group (n = 20): iWhite Instant (Sylphar N. V., Deurle, Belgium) without bleaching agents (ingredient of the placebo agent: aqua, glycerin, sorbitol, potassium acesulfame, chondrus crispus powder, hydrated silica, aroma, methyl paraben, anti-oxidant).

**Figure 1:**
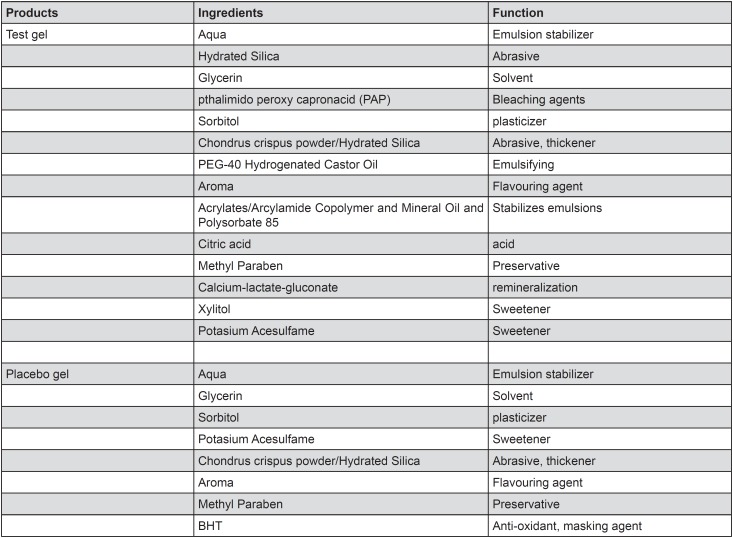
Ingredients of the test and placebo bleaching gels

The ingredients of the products used in this study are listed in Figure 1. The products had the same packaging.

The key list that documented to which group the subjects belonged was kept by a third person and was not revealed to the clinical examiner until the study was completed. No participant indicated the presence of a systemic disease in his/her medical history. An intraoral examination confirmed that each participant had an age between 18-65 years, at least 22 natural teeth with no current caries activity, gingivitis, periodontal disease, or other oral pathology. The front teeth should not be lighter than A2 and without defective enamel structure or restorations. Exclusion criteria were the presence of any systemic disease, pregnancy or breastfeeding, the use of fixed or removable orthodontic appliances, smoking, alcohol abuse, allergic reaction to any component of the agents, the use of any bleaching agents within the last year and dental hypersensitivity. Subjects were instructed to use the same non-whitening toothpaste (Sensodyne Fluoride, GlaxoSmithKline Consumer Healthcare GmbH & Co. KG, Hamburg, Germany) and a soft toothbrush (Sensodyne Precision; GlaxoSmithKline Consumer Healthcare GmbH & Co. KG; Hamburg, Germany) throughout the experiment. Whitening products and the consumption of curry, coffee, black tea or red wine were strictly forbidden during the study.

### Calibration of the examiner

All examination procedures and subject instructions were performed by the same experienced clinician (JD) to avoid interexaminer differences. Before the study, the clinician was calibrated on 20 subjects to ensure the validity of the tooth color measurements using the VITA shade guide (Vita Zahnfabrik, Bad Säckingen, Germany). The second tooth color measurements were performed after a two-week break. The strength of agreement was assessed by calculating Kappa values. The intraexaminer correlation coefficient was 0.83; hence, the strength of agreement can be rated as almost perfect.

### Examination

Efficacy (tooth color) and safety (side effects such as tooth sensitivity and gingival irritation) were assessed at baseline (E_0), immediately after treatment (E_1) and 24 h after treatment (E_2). Tooth color was measured using the VITA Lumin shade guide (Vita Zahnfabrik, Bad Säckingen, Germany), which consists of the 16 most common tooth colors (1-16). The examiner assessed patients’ conditions by deploying a blast of air to teeth isolated with cotton rolls, from a distance of 1 cm, for 1 s. The scale was used as follow: 0 = absence of pain and 1 = presence of pain (slight, moderate and severe sensitivity). Gingival irritation, i.e. any abnormal findings such as redness, edema or epithelial irritation of soft tissues was recorded by the examiner. The next parameters were self-reports for tooth sensitivity and gingival irritation at the final examination. Tooth sensitivity included pain after the consumption of cold and hot beverage and/or food respectively and gingival irritation involved any discomfort or gingival irritation. The scale recorded as 0 = absence of pain and 1 = presence of pain.

Treatments were performed according to the manufacturers’ instructions. The test and placebo gels were placed on a ready-to-use tray for six upper and six lower anterior teeth. The examiner supervised the subjects to ensure correct application. They were not allowed to remove the trays during the treatment session but they were allowed to talk. After 20 min, the trays were removed and discarded. Teeth were cleaned from remnants of the gel. Thereafter, the tooth color, tooth hypersensitivity and the condition of the soft tissue were recorded. The fluoride gel (Elmex Gelée, CP GABA, Hamburg, Germany) was applied subsequently for five minutes. The final examination was performed after 24 h.

The individual color determinations were evaluated by one examiner under identical conditions using the VITA Lumin shade guide (Vita Zahnfabrik, Bad Säckingen, Germany). The VITA shade guide served as reference to the clinical situation and consists of the 16 most common tooth colors sorted according to shade^5^ ^,^ ^18^. The middle one-third of the facial surface of the upper and lower anterior teeth was measured to select the shade. A single average shade score of upper and lower anterior teeth was calculated for each individual.

A clinical examination of the oral and perioral region was conducted to reveal any signs of adverse changes to teeth or soft tissue after treatment, in addition to the questionnaires at the final examination. Tooth hypersensitivity and oral irritation were the most important issues. These represent the most common adverse events associated with vital bleaching^9^. The severity and duration of adverse events were recorded.

### Statistical analysis

Statistical analyses were performed by statistical software (SPSS for Windows version 20, Superior Performing Software Systems, Chicago, USA). For each group, mean teeth color shade at different times of examination was calculated with standard deviation and 95% confidence intervals. The Kolmogorov- Smirnov test showed an inhomogeneous distribution of the data. We applied non-parametric tests due to the asymmetric data distribution. The non-parametric Mann-Whitney U test was used for analysis. The significance level was set at *p*<0.05. The analysis of the secondary data was carried out in a descriptive manner.

## Results

Forty subjects between 18 and 64 years participated in this study. Baseline demographic and clinical characteristics for each group are shown in Table 1.

**Table 1:** Distribution of subjects by gender, age, and VITA color scale at baseline examination

	Female		Male		Age		VITA color scale	
	n	%	n	%	mean	SD	mean	SD
iWhite Instant	12	60	13	65	34.76	13.54	9.837	1.928
Placebo	8	40	7	35	38.02	13.90	9.208	1.114

**Figure 2:**
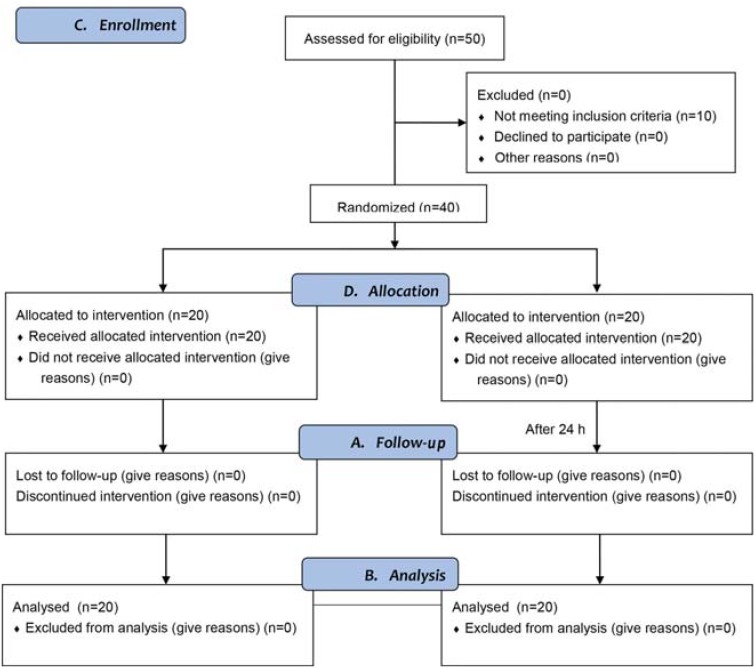
CONSORT 2010 flow diagram

The dropout quote was 0%. No significant difference in color was found between groups at baseline examination. After a single-use bleaching, the tooth color shade for baseline showed a significant difference between iWhite and the placebo immediately and 24 h after treatment (Figure 2).

The mean (±standard deviation, and 95% confidence level, 95% Cl) tooth color shade E_0 was 9.84 (±1.93, 95% CI=8.94-10.74) unit for the test group and 9.21 (±1.11, 95% CI=8.69-9.73) unit for the placebo group. The mean tooth color shade E_1 was 7.58 (±1.66, 95% CI=6.80-8.36) unit for the test group and 9.22 (±1.16, 95% CI = 8.68-9.76) unit for the placebo group. The mean of tooth color shade E_2 was 7.79 (±1.73, 95% CI=6.78-8.60) unit for the test group and 9.31 (±1.01, 95% CI = 8.84-9.79) unit for the placebo group.

**Figure 3:**
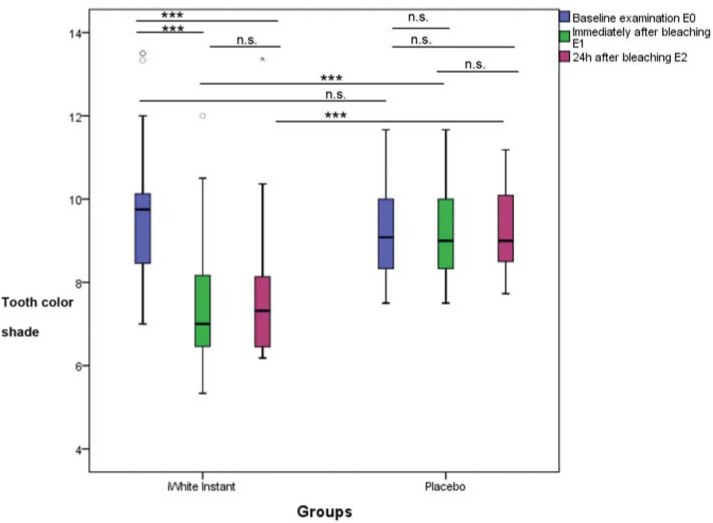
Values of the tooth color of the bleaching product and the placebo for baseline examination, immediately and 24 h after bleaching; n.s. (not significant); *** (p<0.001)

The tooth color shade change for the placebo group showed no significant differences between baseline and immediately after bleaching (ΔE1_0), between immediately and 24 h after bleaching (ΔE1_2), and between baseline and 24 h after bleaching (ΔE2_0) (Figure 3). The test group showed significant differences for ΔE1_0 and ΔE2_0 but not for ΔE1_2.

**Figure 4:**
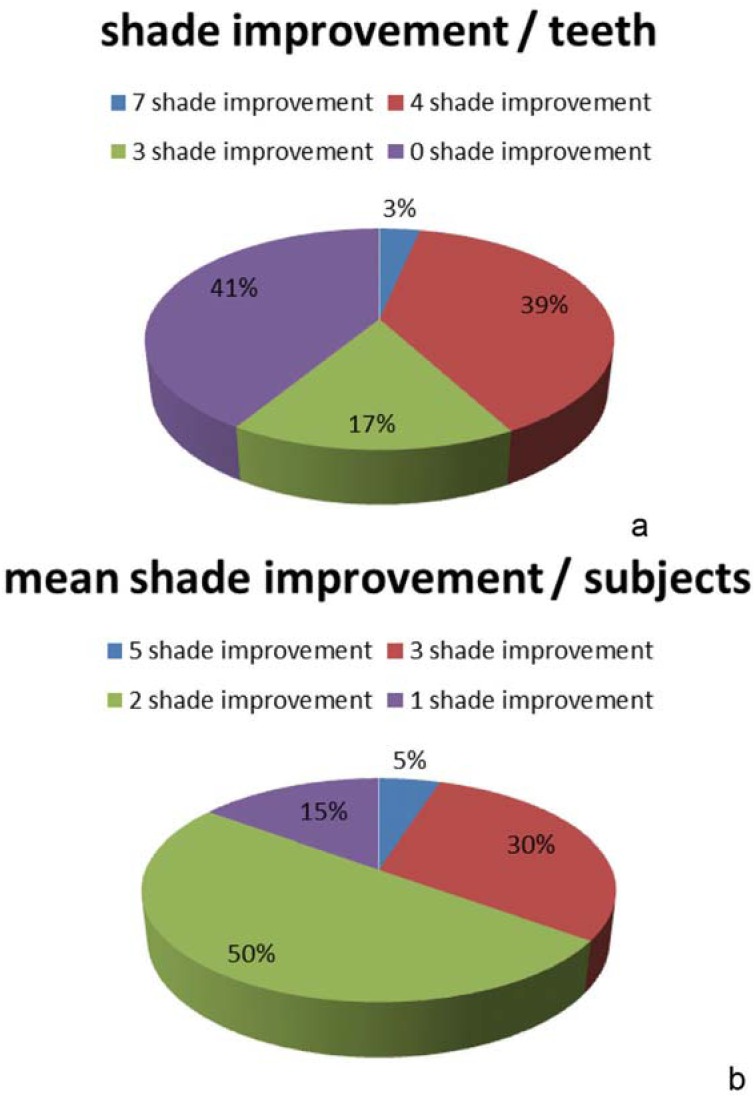
Distribution of shade improvement for the iWhite Instant between baseline and immediately after bleaching (a) *per* teeth (b) *per* subjects

In the test group, 39% of the examined teeth had a shade improvement of four shade values immediately after treatment, 17% had an improvement of three shades, and 3% had an improvement of seven shades. Among the examined teeth, 41% showed no shade value improvement (Figure 4a). When assessed *per* individual, 5% of the test group showed an average improvement of five shades, 30% had an average improvement of three shades, 50% an average of two shades, and 15% an average of one shade (Figure 4b).

The examiner observed two subjects (immediately after treatment) and three (24 h after treatment) with gingival irritation on soft tissue during the study in the test group. None of the subjects had dental hypersensitivity. Three subjects in the placebo group were diagnosed with hypersensitivity and three with gingival irritation (immediately after treatment) (Table 2).

**Table 2:** Development of tooth hypersensitivity and gingival irritation after immediate (E_1) and final examination after 24 h (E_2)

Examiner	iWhite Instant (E_1)		iWhite Instant (E_2)		Placebo (E_1)		Placebo (E_2)	
	n	%	n	%	n	%	n	%
No hypersensitivity	20	100	20	100	17	85	20	100
hypersensitivity	0	0	0	0	3	15	0	0
No gingival irritation	18	90	17	85	17	85	18	90
Gingival irritation	2	10	3	15	3	15	2	10
**Self-report**	**iWhite Instant (E_2)**				**Placebo (E_2)**			
	**n**		**(%)**		**n**		**(%)**	
No hypersensitivity	19		95		20		100	
Hypersensitivity	1		5		0		0	
No comment	0		0		0		0	
No gingiva irritation	17		85		17		85	
Gingiva irritation	3		15		0		0	
No comment	0		0		3		15	

Two subjects in the test group reported gingival irritation, one subject gingival edema immediately after treatment and one subject reported tooth hypersensitivity immediately after treatment. These adverse events had disappeared after 24 h (Table 2). In the placebo group, three subjects reported "no comment" instead of "no gingival irritation" or "gingival irritation".

## Discussion

Bleaching is an oxidative process that alters the light absorption or reflection on the tooth surface. The bleaching treatment is intended to be part of a complete dental procedure. The dentist's examination is necessary to ensure the correct indications for bleaching, and he/she monitors the treatment to avoid significant side effects^19^. A double-blind placebo- controlled clinical study was conducted to evaluate initial color improvement after a single-use application of the newly marketed non-peroxide bleaching system. The results of our study demonstrated that the nonperoxide bleaching system is effective.

Gerlach and Zhou^13^ (2001) showed that the more yellow the teeth at the baseline the better the outcome of the tooth bleaching^13^. Therefore, the bleaching groups were balanced for baseline tooth color and age. The test bleaching products may potentially cause irreversible damage if used on a long-term basis; so we decided to perform a single-use application under the supervision of a dentist.

The tooth color alterations were assessed using a shade guide and the visual evaluation from one examiner, which is in accordance with other clinical studies^6^ ^,^ ^22^. Visual inspection is a clinical subjective method^17^, therefore the Kappa coefficient value for intraexaminer reliability was assessed before the start of the study (0.83) - ensuring a high reliability of the evaluated tooth colors^25^. Interference by surrounding light is a disadvantage for the color determination by means of a shade guide. The color impression of a tooth depends on the translucency, the reflection and the adsorption of the light on the tooth surface. The tooth color was examined under standardized light and surrounding influences to eliminate artefacts.

The investigated novel bleaching agent (iWhite Instant) consists of phthalimido peroxy caproic acid (PAP), an organic peracid containing a high potential of oxidation. Oxidation is necessary for the bleaching procedure as it neutralizes organic double bonds that cause dental discolorations. But the activity of PAP is not based on the release of hydrogen peroxide^11^. Risks involved in at-home and in-office bleaching procedures are tooth hypersensitivity and gingival irritations. These side effects depend on the concentration of the bleaching agent and the contact between the tray and the gingiva^9^. Therefore, a mucosal irritation or dental hypersensitivity may also occur after the use of non-peroxide bleaching agents. No dental hypersensitivities and mucosal irritations were observed by the examiner immediately and 24 h after the single use of the new bleaching agent. Fluoride was applied after the treatment which may also have a remineralizing effect on the enamel^7^. But self-reported dental hypersensitivity and mucosal irritation indicate that these side effects have to be examined in long-term studies.

**Figure 5:**
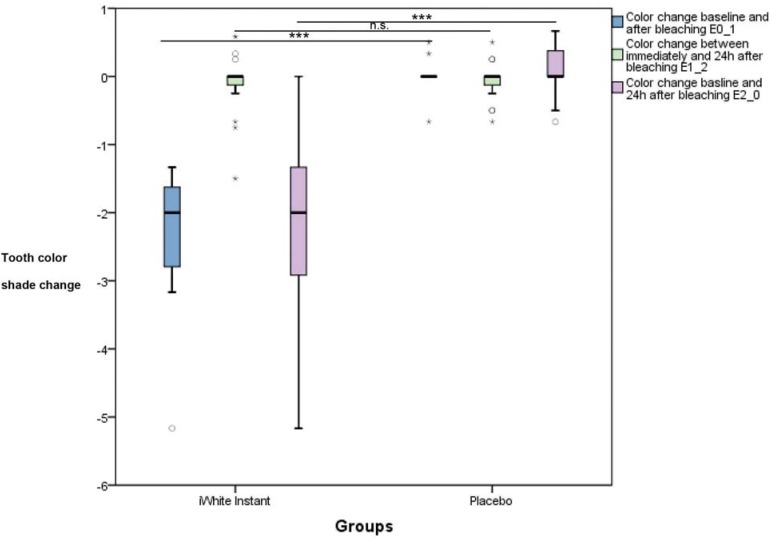
Values of the color change for bleaching and placebo groups between baseline and immediately after bleaching, between immediately and 24 h after bleaching, and between baseline and 24 h after bleaching n.s. (not significant); *** (p<0.001)

There was a significant difference between the active bleaching agent and the placebo. Tooth whitening using at-home OTC bleaching agents has already been examined in several studies^6^ ^,^ ^16^ ^,^ ^26^ and was shown to be effective for weak tooth discolorations^16^. In this study, 59% of all examined teeth between baseline and immediately after bleaching showed an improved color shade in the test group (Figure 5). Nevertheless, not all teeth were homogenously bleached depending on the position and individual characteristics like discoloration pattern or anatomic structure. The ready-to-use tray may also be a reason for irregular bleaching results and gingival irritation. The color stability after bleaching has been largely confined to weeks or months^2^ ^,^ ^6^ ^,^ ^28^.

The bleaching effect is well known and depends not only on the bleaching method but also on subjects’ food and lifestyle habits^21^. We investigated the short-term effect of the novel OTC non-peroxide bleaching agent 24 h after bleaching. No significant color change was observed within 24 h after bleaching. Further studies about the effectiveness after 14 days of the application and a long-term color stability after months are needed for the non-hydrogen peroxide bleaching agent.

The single use and the short-term observation for the effect of color stability were the limitations of this study, which, on the other hand, were necessary to assess the initial effectiveness of the new bleaching agent. The single application of the novel product showed a significant effect which was not yet reported for hydrogen peroxide products.

## Conclusions

Within the limitations of this study, the results showed that single-use bleaching containing a phthalimido peroxy caproic acid (PAP) agent yielded significant initial whitening compared to baseline and placebo. These results remained stable in time, e.g. 24 h after application.

## References

[1] Addy M, Moran J (1985). Extrinsic tooth discoloration by metals and chlorhexidine. II. Clinical staining produced by chlorhexidine, iron and tea. Br Dent J.

[2] Al Shethri S, Matis BA, Cochran MA, Zekonis R, Stropes M (2003). A clinical evaluation of two in-office bleaching products. Oper Dent.

[3] Alkhatib MN, Holt R, Bedi R (2004). Prevalence of self-assessed tooth discolouration in the United Kingdom. J Dent.

[4] American Dental Association Council on Scientific Affairs (2009). Tooth whitening/bleaching: treatment considerations for dentists and their patients [internet].

[5] Auschill TM, Hellwig E, Schmidale S, Sculean A, Arweiler NB (2005). Efficacy, side-effects and patients’ acceptance of different bleaching techniques (OTC, in-office, at-home). Oper Dent.

[6] Bizhang M, Müller M, Phark JH, Barker ML, Gerlach RW (2007). Clinical trial of long-term color stability of hydrogen peroxide strips and sodium percarbonate film. Am J Dent.

[7] Bizhang M, Seemann R, Duve G, Römhild G, Altenburger JM, Jahn KR (2006). Demineralization effects of 2 bleaching procedures on enamel surfaces with and without post-treatment fluoride application. Oper Dent.

[8] Borges AB, Torres CR, Souza PA, Caneppele TM, Santos LF, Magalhães AC (2012). Bleaching gels containing calcium and fluoride: effect on enamel erosion susceptibility. Int J Dent.

[9] Bruzell EM, Pallesen U, Thoresen NR, Wallman C, Dahl JE (2013). Side effects of external tooth bleaching: a multi-centre practice-based prospective study. Br Dent J.

[10] Demarco FF, Meireles SS, Masotti AS (2009). Over-the-counter whitening agents: a concise review. Braz Oral Res.

[11] Fässler M, Meissner K, Schneider A, Linde K (2010). Frequency and circumstances of placebo use in clinical practice - a systematic review of empirical studies. BMC Med.

[12] Faul F, Erdfelder E, Buchner A, Lang AG (2009). Statistical power analyses using G*Power 3.1: tests for correlation and regression analyses. Behavior Res Methods.

[13] Gerlach RW, Zhou X (2001). Vital bleaching with whitening strips: summary of clinical research on effectiveness and tolerability. J Contemp Dent Pract.

[14] Goldberg M, Grootveld M, Lynch E (2010). Undesirable and adverse effects of tooth-whitening products: a review. Clin Oral Investig.

[15] Hasson H, Ismail AI, Neiva G (2006). Home-based chemically-induced whitening of teeth in adults. Cochrane Database Syst Rev.

[16] Horn BA, Bittencourt BF, Gomes OM, Farhat PA (2014). Clinical evaluation of the whitening effect of over-the-counter dentifrices on vital teeth. Braz Dent J.

[17] Judeh A, Al-Wahadni A (2009). A comparison between conventional visual and spectrophotometric methods for shade selection. Quintessence Int.

[18] Leonard RH, Bentley C, Eagle JC, Garland GE, Knight MC, Phillips C (2001). Nightguard vital bleaching: a long-term study on efficacy, shade retention. side effects, and patients’ perceptions. J Esthet Restor Dent.

[19] Li Y (2011). Safety controversies in tooth bleaching. Dent Clin North Am.

[20] Majeed A, Grobler SR, Moola MH, Oberholzer TG (2011). Effect of four over-the-counter tooth-whitening products on enamel microhardness. SADJ.

[21] Matis BA, Cochran MA, Franco M, Al-Ammar W, Eckert GJ, Stropes M (2007). Eight in-office tooth whitening systems evaluated *in vivo*: a pilot study. Oper Dent.

[22] O'Brien WJ, Groh CL, Boenke KM (1989). One-dimensional color order system for dental shade guides. Dent Mater.

[23] Pintado-Palomino K, Tirapelli C (2015). The effect of home-use and in-office bleaching treatments combined with experimental desensitizing agents on enamel and dentin. Eur J Dent.

[24] Scientific Committee on Consumer Products (European Commission) (2005). Opinion on hydrogen peroxide in tooth whitening products [internet].

[25] Sim J, Wright CC (2005). The kappa statistic in reliability studies: use, interpretation, and sample size requirements. Phys Ther.

[26] Soparkar P, Rustogi K, Zhang YP, Petrone ME, DeVizio W, Proskin HM (2004). Comparative tooth whitening and extrinsic tooth stain removal efficacy of two tooth whitening dentifrices: six-week clinical trial. J Clin Dent.

[27] Xiao J, Zhou XD, Zhu WC, Zhang B, Li JY, Xu X (2007). The prevalence of tooth discolouration and the self-satisfaction with tooth colour in a Chinese urban population. J Oral Rehabil.

[28] Zekonis R, Matis BA, Cochran MA, Al Shetri SE, Eckert GJ, Carlson TJ (2003). Clinical evaluation of in-offíce and at-home bleaching treatments. Oper Dent.

